# Change in the Use of Electroconvulsive Therapy Between 2012 and 2022 in Northern Finland

**DOI:** 10.1097/YCT.0000000000001275

**Published:** 2026-06-03

**Authors:** Matias Leppäaho, Saana Karttunen, Marianne Haapea, Anna-Sofia Myllynen, Helinä Hakko, Erika Jääskeläinen

**Affiliations:** * From the Department of Psychiatry, Oulu University Hospital and Wellbeing Services County of North Ostrobothnia; † Research Unit of Population Health, University of Oulu; ‡ Medical Research Center Oulu, Oulu University Hospital and University of Oulu, Oulu, Finland

**Keywords:** ECT, electroconvulsive therapy, usage

## Abstract

**Objectives:**

The demand for Electroconvulsive Therapy (ECT) has been increasing. The aim of this study was to analyze changes in the rate of ECT use at Oulu University Hospital in Northern Finland from 2012 to 2022 and to explore factors associated with these changes.

**Methods:**

The data include all patients who received ECT between April 2012 and the end of 2022 at the neuromodulation unit in the department of psychiatry at Oulu University Hospital. The unit’s catchment area covers a population of 484,400 people. The study focuses on the annual number of ECT and maintenance ECT (M-ECT) sessions, the ECT use rate per 100,000 people, and the number of individual patients and their characteristics, with a focus on yearly changes.

**Results:**

The number of ECT sessions has increased significantly during the follow-up period. There were 1373 ECT sessions in the first full year of 2013 and 3000 sessions in 2022. The growth was particularly notable in M-ECT. ECT was performed on 96 unique patients in 2013 and 189 in 2022. The ECT use rate in 2022 was 45.7 per 100,000 population. 54.6% of the patients were female. The most common diagnosis was major nonpsychotic depression (45.9%).

**Conclusions:**

The results show a steady increase in the demand for ECT. The use rate in Northern Finland is higher than in the previous Finnish study and among the highest in Europe. Improved strategies to prevent post-ECT relapses and alternative treatment methods for treatment-resistant depression (TRD) are needed.

## BACKGROUND

Depression is a major global health burden. It has an estimated global prevalence of 3.8%.^1^ Depressive disorders were ranked second in Years of healthy life lost due to disability (YLD) in the World.^2^ One in 10 adults in Finland suffered from a depressive disorder (major depressive disorder or dysthymia) in 2011.^3^ Depression significantly reduces the quality of life and was the leading cause of early retirement in Finland in 2019. 33% of people receiving disability pension had a mental health problem, of which depression is the most common cause.^4^ Major depressive disorder (MDD) is also highly recurrent—nearly two-thirds of patients experience at least 1 recurrent episode. The risk of recurrence was estimated to increase by 16% after each recurrent episode.^5^

Depending on the definition and study approach, 4% to 21% of patients suffer from treatment-resistant depression (TRD).^6^ The most common definition of TRD is failure to respond (at least 50% decrease in MADRS-score) to 2 adequate (4 to 6 wk) courses of antidepressant medication.^7^ In a recent Finnish nationwide cohort study, the prevalence of TRD is estimated to be 11%.^8^ The overall expenditure these patients have in Finland’s economy is nearly double that of regular depression.^9^ All-cause mortality was 17% higher, and the suicide rate was 90% increased in TRD compared with non-TRD in Finland.^10^

The treatment of depression is often inadequate in Finland. Even in the fifth line, the most common pharmacological treatment is still antidepressant monotherapy.^8^ Neuromodulation therapies are considered options reserved for TRD cases. Electroconvulsive therapy (ECT) is widely regarded as the most effective treatment for depression, although intravenous ketamine has shown noninferiority.^11^ Commonly available psychiatric neuromodulation therapies in Finland include repeated transcranial magnetic stimulation (rTMS), transcranial direct current stimulation (tDCS), intravenous ketamine infusion, and ECT. ECT has been administered in Finland on a larger scale since 1944, but its use has been limited by availability and general attitudes. ECT has generally been portrayed negatively and inaccurately in the media.^12^ The mean waiting time to receive ECT was 26.5 days in a 2013 Finnish ECT survey,^13^ and a similar survey from 2024 showed that in 57% of Finnish units providing ECT, the waiting time for a non-acute course was 1 to 3 months.^14^ Our clinical practice supports this finding of steadily growing queues.

We have noticed a significant increase in the demand for ECT over time in our clinical work. The change has not been defined, for example, whether it concerns only maintenance-ECT(M-ECT) or acute series. Previous national surveys have examined the practice of ECT.^13^,^15^,^16^,^17^,^18^,^19^,^20^,^21^,^22^,^23^,^24^,^25^ The ECT use rate in these previous surveys ranged from 1.76 to 43.7 patients/100,000 people. These studies mainly focus on a single year or are compared with a previous survey. Danish studies have shown an increasing number of ECTs.^15^,^25^ The magnitude of change in use and the factors related to it have not been studied before over a several-year follow-up period. This study provides important insights into the relationship between ECT patient characteristics and changes in its use. It is important to study the differences in ECT use to identify geographical variations and changes over time as new forms of treatment emerge. National guidelines, access to ECT, stigma, and cultural attitudes can affect the usage.

This study aimed to evaluate the magnitude of change in ECT use in Northern Finland between 2012 and 2022. Another aim was to study the associations between patient characteristics and the change over time in the use of ECT.

## MATERIALS AND METHODS

### Materials

Oulu University Hospital’s psychiatric neuromodulation unit is located in the Wellbeing services county of Northern Ostrobothnia (formerly Northern Ostrobothnia hospital district until Dec 31, 2023), which has a population of ∼400,000 living in 30 municipalities. The unit also provides ECT for nearby Wellbeing services in the county of Kainuu (Kainuu hospital district until Dec 31, 2023), with a population of ∼74000 people. The unit has consistently been the only provider of ECT in these catchment areas during the research period. A particular characteristic of these areas is an elevated prevalence of psychosis compared with the rest of Finland. For example, between 2013 and 2022, the percentage of people receiving reimbursement for antipsychotics ranged from 1.9 to 2.1 in Northern Ostrobothnia compared with 1.7 to 1.9 in the rest of Finland. The proportions of people aged 18 to 64 per 1000 people of the same age receiving reimbursement for antidepressants ranged from 73 to 94 and 76 to 97, respectively.^26^ Besides ECT, there are also rTMS, tDCS, and ketamine infusions available in the neuromodulation unit.

The data analyzed in this study comprise all visits of patients treated with ECT from the foundation of the neuromodulation unit in April 2012 to the end of 2022. The data on ECT visits were collected from the electronic patient register of Oulu University Hospital. The search was narrowed down to only ECT visits by using a procedure code (NCSP, Nordic Classification of Surgical Procedures) of IFB01 ECT, AA400 ‘Electric shock as a treatment of psychiatric illness, or cases with no procedure code. The sample comprised patients receiving ECT as both outpatients and inpatients. Before analyses, the data were manually checked and corrected for errors and discrepancies by the main researcher (ML).

### Treatment Practice of Electroconvulsive Therapy From 2012 to 2022

In the neuromodulation unit's clinical practice, patients are referred to ECT by a physician and evaluated for eligibility during an outpatient visit by a psychiatrist or a resident psychiatrist. The evaluation focuses mainly on the indications, contraindications, and explaining the content and purpose of ECT to the patient. The diagnoses marked by the referring physician in the referral to ECT are generally used throughout the treatment course. Indications for ECT include treatment-resistant depression (defined as failure of at least 2 adequate courses of antidepressant medication), clozapine-resistant schizophrenia, catatonia, treatment-resistant mania, or bipolar depression. Acute or untreated substance-use disorder (ICD-10 codes: F10-F19) has typically been considered a contraindication for the treatment, but other secondary psychiatric diagnoses have been included. Good prognostic features (such as higher age, psychotic or melancholic depression) have favored the clinician’s choice of ECT over other types of neuromodulation therapies. After evaluation of the patient’s eligibility for ECT, a course of ECT has been prescribed, typically thrice weekly for a total of 12 treatment sessions. From 2019 onwards, ECT courses for psychotic disorders have been prescribed to include at least 15 treatment sessions.

Electroconvulsive therapy (ECT) has been delivered with the Somatics Thymatron System IV device using a 2x dose stimulus program, which allows a maximum of 1008 mC of energy and automatically adjusts the pulse width and frequency to obtain the longest possible duration for any given percent energy dial setting. The initial dose has been calculated using a half-age-based method (½x the approximate patient’s age in percent energy dial setting) and adjusted throughout the course based on seizure duration and quality.

From 2012 to 2017, bilateral (BL) electrode placement was the primary method used in clinical practice. Gradually, during 2017, frontoparietal (FP) electrode placement was used as a unilateral form of ECT to reduce side effects. Only right-sided FP ECT was used. FP was the first option for depressed patients, and BL was the first option for patients suffering from psychosis. If there was no sufficient response from FP ECT, the electrode placement could be changed to BL from the sixth treatment session onwards.

Unfortunately, the electronic register data on outpatient visits does not include information on different electrode placements or anesthetics.

Electroconvulsive therapy (ECT) has been administered by a health care professional team consisting of a psychiatrist or a resident psychiatrist, a registered psychiatric nurse, a registered nurse in anesthesiology, and an anesthesiologist. Almost all anesthesia has been performed with propofol, succinylcholine, and glycopyrrolate using weight-based dosing. Etomidate has been used as a secondary option if optimal seizure quality could not be achieved.

The patients’ condition, improvement, and side effects have been briefly evaluated before every ECT treatment session by the psychiatrist or the resident psychiatrist. The duration of the ECT course can be adjusted based on the patient’s response, typically shortened if the patient responds quickly to treatment or lengthened to 15 sessions if the response occurs late in the treatment series. The Beck Depression Inventory-21 (BDI-21) was used to measure the patient’s self-reported score before ECT treatment and at the end of the course. M-ECT has been prescribed after the second course if there has been a relapse during the first year after a successful course. Sometimes, if the patient was evaluated to be at a high relapse risk, M-ECT was prescribed straight away. The frequency of M-ECT has been evaluated and adjusted at every treatment session based on symptom level and, more thoroughly, annually via a phone consultation with a psychiatrist or a resident psychiatrist. M-ECT has been discontinued if the patient has been relatively asymptomatic for ∼ a year and the M-ECT interval has been at least 4 weeks. If a patient has experienced heavy, repeated relapses, M-ECT is planned indefinitely.

### Patient Characteristics

We analyzed the patient's sex, age, and diagnosis. The ages of ECT patients were divided into 4 categories (<25, 25 to 44, 45 to 64, and 65 years or older) and reported separately for each year, along with the annual proportions of male and female patients. The 10th revision of the International Classification of Diseases (ICD-10) has been used for recording diagnoses in the electronic patient register since 1996. The diagnoses marked on ECT visits were categorized as unipolar depression: psychotic and nonpsychotic; bipolar disorder: psychotic depression, nonpsychotic depression, and mania/hypomania; psychotic disorders: schizophrenia, schizoaffective disorders, and other psychotic disorders. The remaining diagnoses formed a group. That group also includes diagnoses that are not correct ECT indications, which, for some patients, were in secondary diagnoses and thus not visible in the data. Please see Supplemental Digital File Table 1, Supplemental Digital Content 1, http://links.lww.com/JECT/A384 for ICD-10 codes.

### Statistical Analysis

The annual number of visits and the number of patients who received ECT treatment in the neuromodulation unit are reported for the 11-year period from 2012 to 2022. Time-trend analyses based on regression models focus on the 10-year period from 2013 to 2022, for which complete annual data were available. A Joinpoint regression analysis of trends was used to model time-specific estimates from aggregated data, for example, the number of ECT visits (course, M-ECT) and the number of patients. The aim of this method is to identify the year(s) in which a potential change in the time trend of events occurred. The analysis reports the annual percentage change (APC) in events between trend-change points. Further, the test of parallelism was used to assess whether the time-trend estimates (regression functions) of the ECT course and M-ECT are parallel, allowing different intercepts.^27^ The time trend analyses were performed using the (Joinpoint Regression Program version 5.2.0; National Cancer Institute, Statistical Methodology and Applications Branch, Surveillance Research Program, Bethesda, MD).^27^,^28^

In addition, the sociodemographic characteristics (sex and age) in Table 2 and the main psychiatric diagnosis in Table 3 were examined using information recorded at the last ECT treatment visit during the 11-year study period from 2012 to 2022. This approach enabled us to analyze whether a patient had received only 1 (ie, ECT course) or several ECT (ie, M-ECT) treatment episodes during the entire study period. The percentage distribution over the 10 years was compared between the ECT and M-ECT patient groups, and the analysis was performed using the χ^2^ test. The statistical analyses were performed using (IBM SPSS Statistics version 29.0.1.0 (171); IBM Corp., Armonk, NY).

A language check for this article was performed using the Microsoft Copilot AI tool, based on OpenAI’s GPT-4, in a protected Oulu University environment.

## RESULTS

### Time Trends in Electroconvulsive Therapy Visits and Number of Patients

As shown in Table 1, the total number of ECT sessions during the 10-year period for which complete annual data were available increased from 1373 in 2013 to 3000 in 2022. Specifically, the number of M-ECT sessions and the number of individual patients receiving M-ECT increased from 413 to 1596 and from 28 to 112, respectively, during 2013 to 2022. The number of individual patients who have received ECT series increased only from 83 in 2013 to 113 in 2022. The number of course-ECT-sessions per patient has increased from 9.9 in 2012 to 12.4 in 2022 (Supplemental Digital File Table 2, Supplemental Digital Content 2, http://links.lww.com/JECT/A385).

**TABLE 1:** ECT-sessions and Patients Per Year Between 2012 and 2022

	ECT-sessions (number)			Individual patients (persons)		
Year	Course	M-ECT	Total	Course	M-ECT	Total
2012*	859	142	1001	87	18	87
2013	960	413	1373	83	28	96
2014	1082	345	1427	101	26	114
2015	1504	588	2092	138	46	158
2016	1323	779	2102	113	58	155
2017	1362	820	2182	120	57	155
2018	1388	969	2357	113	68	157
2019	1380	1149	2529	111	75	162
2020	1539	1246	2785	127	91	180
2021	1512	1535	3047	124	100	192
2022	1404	1596	3000	113	112	189
Total†	14313	9582	23895	986	271	987

A course refers to a prescribed course or a series of ECT sessions.

* Since the neuromodulation unit's services began in April 2012, the numbers cover a 9-month period from April to December 2012.

† The total number of individual patients is calculated separately for each follow-up year as well as over the whole 11-year study period. The number of individual patients over the whole study period is smaller than the sum of individual patients over each year, because an individual patient can have received ECT treatment during multiple years of the study period. M-ECT indicates Maintenance-ECT.

**FIGURE 1:**
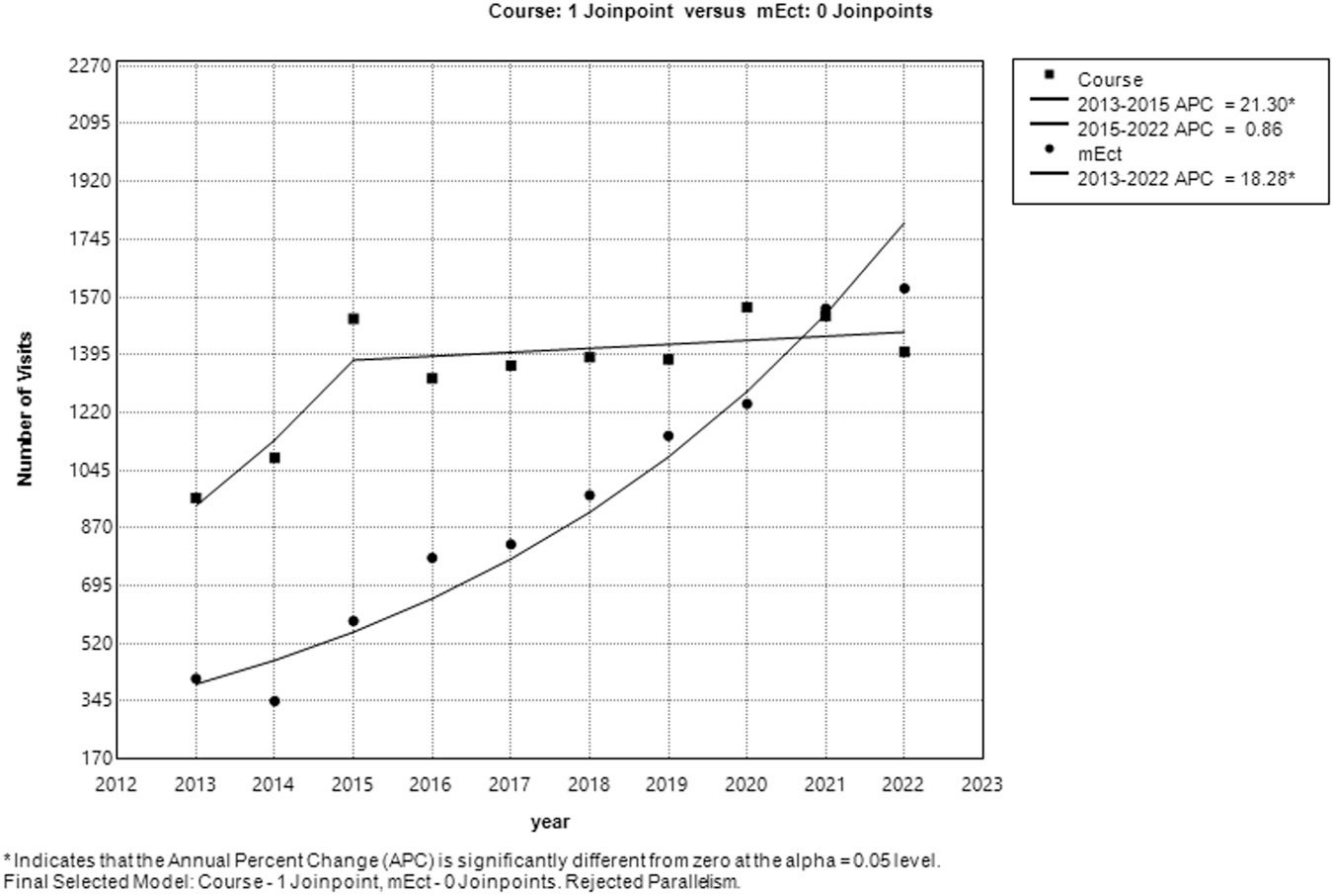
A Joinpoint trend analysis of the number of sessions in the ECT-course and maintenance ECT-sessions between 2013 and 2022.

Figure 1 shows the 10-year time trends in ECT visits to the neuromodulation unit, by course and M-ECT type. The ECT visits (course) had significantly increased from 2013 to 2015 (Annual Percentage Change, APC=21.30, 95% CI for APC=3.19 to 42.58, *P*=0.028), but stabilized from the year 2015 onwards (APC=0.86, −1.30 to 3.06, *P*=0.356), while M-ECT visits had increased over the whole study period (APC=18.28, 14.20 to 22.51, *P*<0.001). The analysis rejected the parallelism of the time trends (*P*=0.002).

**FIGURE 2:**
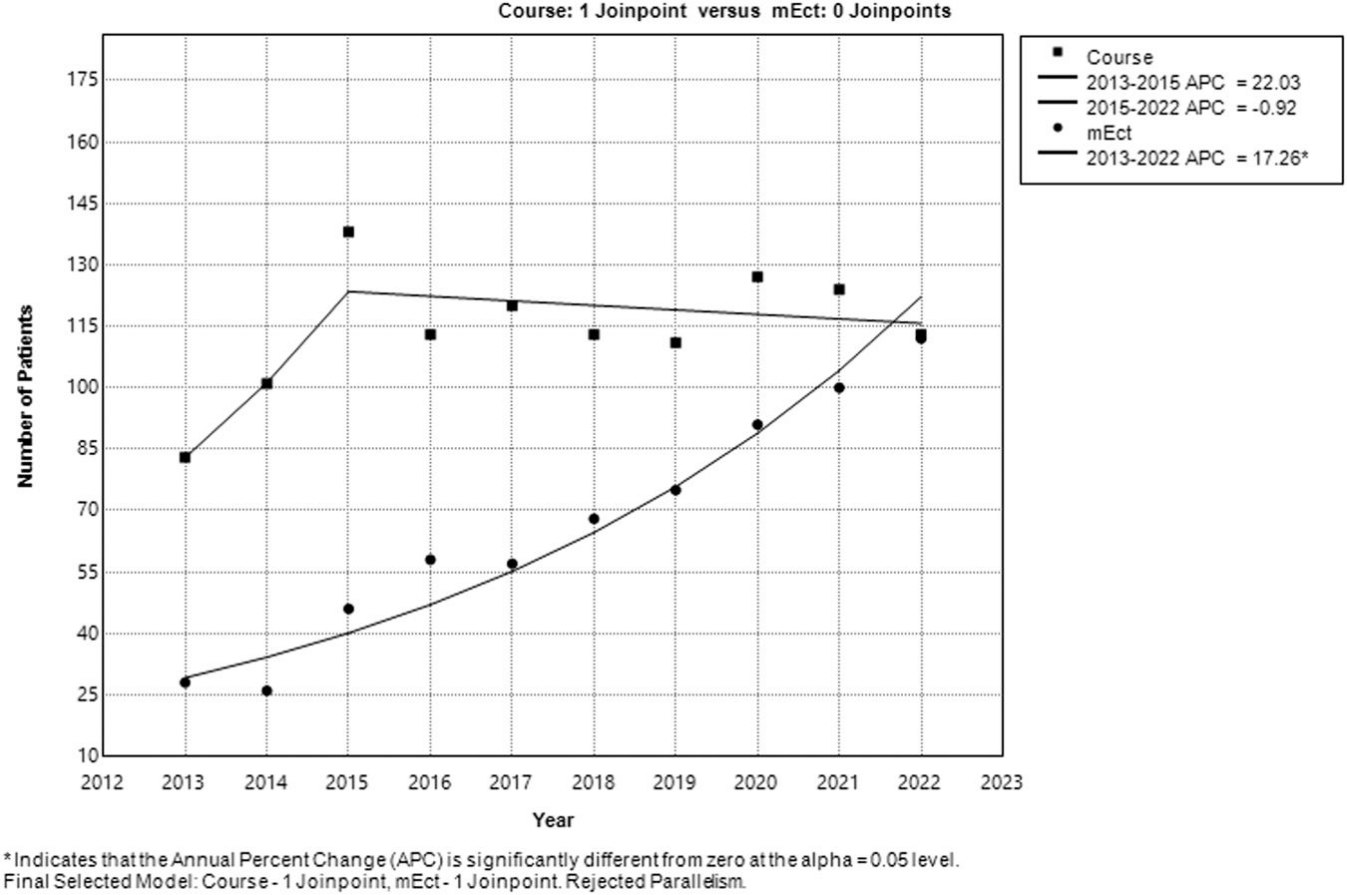
A Joinpoint trend analysis of ECT-patients between 2013 and 2022.

Corresponding 10-year time trends in the number of ECT patients treated in the neuromodulation unit are visualized in Figure 2. The number of ECT patients (per course) showed a marginally significant increase from 2013 to 2015 (APC=22.03, −0.62 to 49.84, *P*=0.055), but decreased slightly from the year 2015 onwards (APC=−0.92, −3.60 to 1.87, *P*=0.426). The number of M-ECT patients, however, showed an increasing trend over the whole study period (APC=17.26, 13.20 to 21.45, *P*<0.001). The difference in these time trends for the number of patients between course and M-ECT visits was statistically significant (test of parallelism, *P*=0.002).

### Age, Sex, and Main Psychiatric Diagnosis of Electroconvulsive Therapy Patients

Further analyses focused on the last ECT treatment visit that occurred during the entire study period (2012 to 2022). Table 2 shows demographics of the patients by the year of their last ECT visit. Of all patients, 54.6% were female, and no statistically significant change in this proportion was observed during the whole study period (χ^2^=14.0, *df*=10, *P*=0.171). The age distribution of the patients differed significantly between the years (χ^2^=60.2, *df*=30, *P*<0.001). Specifically, the annual proportions of patients aged 65 or over fluctuated (range: 10.1% to 37.2%).

**TABLE 2:** Age and Sex of ECT Patients Assessed at Last ECT Visit Occurred During 2012 to 2022 (number of persons and %)

Year of last treatment session	Age group*				Sex	
	<25 (y), n (%)	25-44 (y), n (%)	45-64 (y), n (%)	≥65 (y), n (%)	Men, n (%)	Women, n (%)
2012	3 (5.6)	21 (38.9)	23 (42.6)	7 (13.0)	21 (38.9)	33 (61.1)
2013	2 (3.4)	20 (33.9)	21 (35.6)	16 (27.1)	26 (44.1)	33 (55.9)
2014	8 (11.9)	17 (25.4)	33 (49.3)	9 (13.4)	25 (37.3)	42 (62.7)
2015	7 (7.5)	37 (39.8)	37 (39.8)	12 (12.9)	37 (39.8)	56 (60.2)
2016	5 (5.5)	29 (31.9)	43 (47.3)	14 (15.4)	41 (45.1)	50 (54.9)
2017	8 (9.9)	31 (38.3)	27 (33.3)	15 (18.5)	42 (51.9)	39 (48.1)
2018	7 (8.9)	36 (45.6)	28 (35.4)	8 (10.1)	38 (48.1)	41 (51.9)
2019	5 (6.4)	28 (35.9)	16 (20.5)	29 (37.2)	28 (35.9)	50 (64.1)
2020	9 (9.7)	28 (30.1)	30 (32.3)	26 (28)	52 (55.9)	41 (44.1)
2021	12 (11.7)	40 (38.8)	37 (35.9)	14 (13.6)	53 (51.5)	50 (48.5)
2022	20 (10.6)	54 (28.6)	66 (34.9)	49 (25.9)	85 (45.0)	104 (55)
Total	86 (8.7)	341 (34.5)	361 (36.6)	199 (20.2)	448 (45.4)	539 (54.6)

* The year of the treatment episode and the patient's age refer to the period during the last ECT visit in the study period, 2012 to 2022.

Table 3 and Supplemental Digital File Table 3, Supplemental Digital Content 3, http://links.lww.com/JECT/A386, show the diagnostic categories of the patients based on the main diagnosis set during the last ECT visit over the whole study period. The majority of diagnoses of patients belonged to the diagnostic group of unipolar depression (n=623, 63.1%), followed by psychotic disorders (n=221, 22.4%), bipolar disorders (n=86, 8.7%), and other diagnoses (n=57, 5.7%). This diagnostic distribution differed statistically significantly between the years under analysis (χ^2^=48.0, *df*=30, *P*=0.020). In addition, the most common main psychiatric diagnostic subcategories were major nonpsychotic depression (45.9%), psychotic depression (17.2%), and schizophrenia (12.2%). The proportion of psychotic disorders increased from 10.2% in 2013 to 28.5% in 2022. In contrast, the proportion of unipolar depression decreased from 76.3% to 55.5% accordingly.

**TABLE 3:** Main Diagnostic Categories of Patients at the Last ECT Treatment Episode During the Whole Study Period from 2012 to 2022 (n=987)

**Diagnosis** *	No. patients (% of all patients)
Unipolar depression (n=623)	
Nonpsychotic	453 (45.9)
Psychotic	170 (17.2)
Bipolar disorder (n=86)	
Nonpsychotic depression	65 (6.6)
Psychotic depression	12 (1.2)
Mania, hypomania	9 (0.9)
Psychotic disorders (n=221)	
Schizophrenia	120 (12.2)
Schizoaffective	64 (6.5)
Other	37 (3.7)
Other diagnoses† (n=57)	57 (5.7)

* Diagnostic categories are defined based on the main diagnosis of patients assessed at their last ECT visit during the study period from 2012 to 2022.

† includes diagnoses for OCD (ICD-10: F42.0 to 9), anxiety disorders (ICD-10: F40.0 to 9, F41.1 to 9), PTSD (ICD-10: F43.1) and somatoform disorders (ICD-10: F45.9) (n=24) and rest of diagnosis (n=33).

Table 4 shows the association between patients’ diagnosis and whether the patient has had only a single course of ECT or multiple courses or M-ECT during the entire study period from 2012 to 2022. In total, 644 (65.2%) patients had only a single course of ECT, and 343 (34.8%) patients had multiple courses or were prescribed M-ECT (χ^2^=25.8, *df*=10, *P*=0.004). The majority of patients (53.2%) with schizoaffective disorder needed at least a second course or M-ECT. The percentages regarding schizophrenia were 42.5%, psychotic or nonpsychotic major depression were 35.9% and 30.2%, respectively. The group of other psychotic disorders, including delusional disorder, brief psychotic disorders, and unspecified psychoses, had the lowest proportion of patients in multiple courses or M-ECT (18.9%).

**TABLE 4:** Main Diagnosis Set at Last ECT Treatment Visit of the Individual Patients (n=987) During the Whole 11-year Study from 2012 to 2022, by a Single ECT-course Versus Multiple Courses/M-ECT

Indication*		Only a single ECT-course (n=644), n (%)	M-ECT/multiple courses (N=343), n (%)
Major groups	Subgroups		
Unipolar depression (n=623)	Psychotic (n=170)	109 (64.1)	61 (35.9)
	Nonpsychotic (n=453)	316 (69.8)	137 (30.2)
Bipolar disorder (n=86)	Psychotic depression (n=12)	6 (50)	6 (50)
	Nonpsychotic depression (n=65)	40 (61.5)	25 (38.5)
	Mania, hypomania (n=9)	6 (66.7)	3 (33.3)
Psychotic disorders (n=221)	Schizophrenia (n=120)	69 (57.5)	51 (42.5)
	Schizoaffective disorder (n=64)	30 (46.9)	34 (53.1)
	Other psychotic disorders (n=37)	30 (81.1)	7 (18.9)
Other diagnoses (n=57)	OCD (n=9)	4 (44.4)	5 (55.6)
	Anxiety disorders, PTSD, somatoform disorders (n=15)	12 (80.0%)	3 (20.0)
	Other (n=33)	22 (66.7%)	11 (33.3)

* Diagnostic categories are based on the main diagnosis of a patient assessed at his/her last ECT visit during the whole 11-year study period from 2012 to 2022.

Table 5 shows the change between 2013 and 2022 in the ECT usage rate per 100,000 population and per adult population in Northern Ostrobothnia. The total usage rate in 2013 was 23.8, in 2022, 45.7 patients per 100,000 population, and correspondingly, 31.4 and 58.7 per 100,000 adult population.

**TABLE 5:** ECT Usage Rate Per 100000 People Between 2013 and 2022 in Northern Ostrobothnia

Year	Population*	Adult population†	ECT patients	ECT usage rate/100000 people	ECT usage rate/100000 adults
2013	403555	305491	96	23.8	31.4
2014	405636	305066	114	28.1	37.3
2015	407160	309034	158	38.8	51.1
2016	408296	310305	155	38.0	50.0
2017	409043	312099	155	37.9	49.7
2018	409418	313204	157	38.3	50.1
2019	410112	315376	162	39.5	51.4
2020	411120	316151	180	43.8	56.9
2021	412913	318356	192	46.5	60.3
2022	413907	322019	189	45.7	58.7

* Total population of the Hospital district of Northern Ostrobothnia at the end of the year (Sotkanet Indicator Bank. Finnish Institute for Health and Welfare. Sotkanet.fi. Referenced on 2 May 2025).

† Total population of the Hospital district of Northern Ostrobothnia at the end of the year aged over 18 years (Sotkanet Indicator Bank. Finnish Institute for Health and Welfare. Sotkanet.fi. Referenced on 2 May 2025).

## DISCUSSION

Based on our study and clinical practice observations, the use of ECT has steadily increased from 2013 to 2022. The growth was exponential in maintenance-ECT. Our findings indicate that the number of ECT treatments per patient has increased in both acute courses and M-ECT. The most common diagnostic categories were major nonpsychotic depression, psychotic depression, and schizophrenia.

What causes the growth in demand for ECT? Research suggests that participation in and observation of ECT procedures positively influence medical students’ attitudes toward the treatment.^29^,^30^ At Oulu University Hospital, a voluntary visit to the neuromodulation unit is included in the psychiatry curriculum for medical students. Nursing students frequently visit the unit during their clinical placements. In addition, Finnish media have generally portrayed ECT in a positive light, potentially increasing public and professional interest in the therapy. The rise in ECT usage may also be linked to the long-term reduction in psychiatric hospital bed availability in Finland, including Northern Ostrobothnia. The treatment of patients experiencing difficult symptoms may have shifted more towards neuromodulation therapies instead of psychiatric hospitalization. A report from the Finnish Institute for Health and Welfare shows that there were 22,000 beds in 1970, 7300 in 1993 (66.8% decrease), 4600 in 2015 (37.0% decrease from 1993), 2700 in 2021 (41.3% decrease from 2015).^31^ Between 2013 and 2022, the number of psychiatric hospitalization periods per 1000 people decreased from 7.0 to 6.6, and the hospitalization days from 231.6 to 146.8.^26^

As populations in Finland and other developed countries age—and given that ECT is particularly effective in older adults—this demographic shift may drive increased demand. However, the number of older persons did not increase in our sample during the years, so the aging population does not explain our findings. The number of severely depressed patients may also have increased during the study period, although data about the incidence of depression in Finland during the study years were not available.

In our sample, the proportion of diagnoses of psychotic disorders increased over the years, which may partly explain the increase in usage, as patients with psychotic disorders often require longer treatment courses and are more likely to receive M-ECT. The total increase in ECT usage observed in this study likely reflects a combination of factors: shift in indications, increased knowledge of ECT, increased incidence of depression, and the aforementioned reductions in psychiatric care resources.

### Comparison to Previous Studies

The clinical practice of ECT has been surveyed in Finland by Sumia and others in 2021^12^ and in many other countries. National surveys show variations in clinical practice, usage rates, availability, and primary diagnoses. In Europe, the highest rate of use (41 per 100,000 population) is in Sweden^20^ and Belgium,^19^ and lowest in Poland (1.3/100,000)^21^ and Hungary (1.8/100,000).^22^ The majority of ECT-patients are reported to be female, with a prevalence ranging from 59% (Finland)^13^ to 67% (UK).^18^ Only in Thailand, women were the minority (28%) among ECT patients.^17^

In Sumia's survey,^12^ the main diagnosis was nonpsychotic depression, which is in line with our study and with other Western European countries.^23^,^24^,^25^ In Eastern Europe, the percentage of psychotic disorders tends to be higher.^21^,^22^

Previous studies have mainly analyzed nationwide ECT use using single-year data, and longitudinal analyses of annual trends are scarce. Exceptions include Danish studies^15^,^25^ that used the National Patient Register (DNPR) and show that the number of ECT sessions and unique patients increased from 2008 (18,573 sessions and 1685 patients) to 2017 (21,730 and 1891, respectively). The magnitude of the increase can be considered smaller than in our study. Across Denmark as a whole, the increase was 17% from 2008 to 2017, compared with 118% in Northern Ostrobothnia over a similar period from 2013 to 2022.

In our study, the ECT use rate in 2022 was 45.7/100,000 population, which is higher than the rate previously reported by Sumia et al.^13^ They reported a regional ECT usage of 23.7 patients/100,000 population of Northern Ostrobothnia in 2013, which was slightly above the national mean of 23/100,000 population. In our sample, the usage rate in 2022 increased significantly from 2013, but no national comparison for 2022 is available due to the lack of more recent studies or a national ECT quality registry.

The increase in usage, among the highest in Europe, can be considered a positive development, as it may reflect improved availability of ECT in the area. However, the large number of M-ECT sessions raises important questions. Is patient selection appropriate? Is the acute ECT-course administered correctly, and could a tapering of the course reduce C- or M-ECT? The steady increase in M-ECT suggests that patients remain dependent on ECT after the acute course. That dependence could be reduced by treating depressive and psychotic disorders more efficiently with medication and psychosocial treatments.

### Clinical Discussion

Electroconvulsive therapy (ECT) is an effective treatment method and has a variety of uses. ECT is considered superior in efficacy compared with antidepressant drugs^32^ and in comparison with ketamine in the acute phase of a major depressive episode.^33^ ECT is particularly effective in psychotic depression and elderly patients.^34^ ECT is also associated with lower 1-year all-cause mortality and a reduced short-term suicide risk in older patients.^35^

Other common indications for ECT are catatonia, schizophrenia, bipolar depression, and mania. At a group level, ECT is less effective in schizophrenia than antipsychotic medication.^36^ However, ECT still holds a specific role in the treatment of schizophrenia. Almost 50% of patients not responding to clozapine responded to a combined 8-week course of ECT.^37^

In both psychotic and affective disorders, relapses after a successful course of ECT are a major problem. Pharmacotherapy, continuation-ECT, or maintenance-ECT are viable in the prevention of relapse. C-ECT can be defined as a continuation period of <6 months following an acute course to prevent a relapse of the current illness, and anything over 6 months is considered M-ECT to prevent a new episode of illness.^38^ In previous national surveys, the percentage of patients that had ECT, patients in C-ECT and M-ECT ranged from 14.0%^16^ to 27.1%^13^ but in a recent worldwide survey of ECT practice, as much as a median of 50% of patients who received ECT also underwent C-ECT or M-ECT.^39^

A recent naturalistic cohort study in Denmark by Jørgensen et al^40^ and others showed that C-/M-ECT was associated with a lower rate of hospital readmission after acute ECT and was estimated to be cost-effective. Patients with schizophrenia or schizoaffective disorder were more likely to receive C- or M-ECT. This study had similar results.

Pharmacological strategies have also been studied to prevent post-ECT relapse. In a randomized, placebo-controlled study, the combination of venlafaxine and lithium did not differ in efficacy compared with nortriptyline and lithium. There was no difference in whether to start the medication before or after the acute ECT course. Despite the use of medication, 50% of the patients relapsed, and only 33,6% of patients continued in remission after a 6-month follow-up.^41^ Therefore, it is understandable that C-ECT and M-ECT are necessary, and demand for them has increased over the years.

In a trial^42^ of 290 patients with unipolar major depression, during the 6-month follow-up period, the relapse rate without antidepressant continuation therapy was 84%, and among patients using nortriptyline-lithium therapy, 39%. Relapse rate was higher among medication-resistant patients, female patients, and those with more severe symptoms of depression.^42^ Patients receiving Lithium continuation therapy have been less likely to relapse following a successful ECT course based on a systematic review and meta-analysis.^43^

In future research, it would be important to study further the characteristics of patients who relapse and demand extensive M-ECT. Kramer^44^ and others compared patients with major depression, patients with major depression and a personality disorder, and patients with bipolar disorder in the naturalistic setting of M-ECT. The highest response rate was in major depression and worst in depression with a personality disorder.^44^

In a meta-analysis by Haq et al,^45^ a longer depressive episode predicted a poorer response to ECT. In a randomized clinical trial by Pluijms et al,^46^ the length of the depressive episode was not associated with a relapse within 2 years after successful ECT, but the severity of depression increased the risk. Decreasing waiting times may improve the effectiveness of acute ECT, but the net effect on the total number of treated patients remains difficult to estimate.

Further studies on effectiveness and cost-effectiveness are needed to decide whether to prioritize the limited ECT resources for patients who are more likely to improve and for patients who are in acute danger of suicide. More studies are needed to optimize ECT practice, determine who should receive ECT, and identify the parameters associated with optimal clinical response. Studies focusing on alternative strategies for maintaining remission after ECT are also warranted.

### Strengths

This was the first study in Finland and one of the very few worldwide to show the yearly time trend in ECT use. The data over a 10-year period are unique and provide long-term follow-up information. All the ECT data were collected from the hospital’s patient register, which is a more reliable source than survey data. The relatively high number of ECT sessions and patients, along with a long follow-up period in this real-world setting, can also be considered a notable strength of the current study. Diagnoses related to ECT treatment were analyzed according to ICD-10 diagnostic criteria, thereby enabling international comparison. Finnish healthcare register data has been shown to be satisfactory for research purposes.^47^,^48^

### Limitations

This study was limited to the Northern Ostrobothnia region; therefore, generalizability to the whole country and other parts of the world must be approached with caution. The clinical practice has changed during the follow-up period, which may have affected the annual number of treatments. Unpredictable events, such as the nationwide nurses’ strike in Finland in April 2022, may have affected clinical practice and patient numbers, although the COVID-19 pandemic in 2020 did not affect clinical practice or patient numbers overall. Our data lacked comprehensive information on the proportion of ECT administered to hospitalized patients, which would have provided insight into the severity of the illness requiring inpatient care. The classification of ECT visits to indicate either an acute ECT course or M-ECT was performed by the main researcher. Although our ECT practice follows a protocol, a patient’s received ECT type (acute course or maintenance) can vary depending on current service availability. If a patient’s symptoms increase, M-ECT can, for example, be increased to biweekly for several weeks, and this is labeled as M-ECT instead of reclassifying it as an ECT course.

Although the diagnoses of ECT patients were based on ICD-10 diagnostic criteria, some diagnoses may have remained unspecific. Only the main diagnosis was available in our data set. Comorbid psychiatric diagnoses would have been useful for analysis, as they are known to influence both the efficacy and duration of ECT treatment. Substance use can affect depressive symptoms and is typically assessed through patient interviews and the AUDIT (Alcohol Use Disorders Identification Test). From 2022, we have included blood phosphatidyl ethanol (B-PEth) in pre-ECT laboratory testing to identify excessive alcohol consumption, which may have influenced patient selection in that year. The patients’ medication during or after the ECT was not available in our register data. The mean waiting time from referral to ECT to receiving the first ECT session was also unavailable in this data, although this metric has been included in some national surveys. It could provide important information on the queues and the demand for acute ECT series. The ECT usage rate in the neuromodulation unit's catchment area is an approximation, as the total population comprises 2 Hospital Districts. Some patients from Kainuu may also be referred to other wellbeing service areas. It remains unknown whether, for example, the long distance from Kainuu influences the consideration of ECT as a treatment option.

## CONCLUSIONS

This study showed the magnitude of change in ECT usage at Oulu University Hospital’s neuromodulation unit, which serves a catchment area of 484,000 people. The increase was significant, especially in maintenance-ECT. This study provides important insights into ECT usage and patient characteristics over time. Future studies should focus on optimizing the delivery of ECT and M-ECT and on identifying effective medications for relapse prevention.

## Supplementary Material
