# Supplementary material for: Change in the Use of Electroconvulsive Therapy Between 2012 and 2022 in Northern Finland
Source: J ECT. 2026 Jun 3;42(3):207–15. doi: 10.1097/YCT.0000000000001275 (PMC13561100; doi:10.1097/YCT.0000000000001275)
Supplement: Supplementary file 1 [file yct-42-207-s001.docx]

**Supplemental digital file Table 2.**

Mean number of ECT-sessions in ECT-course and M-ECT per patient

|  | **Number of ECT-sessions/patient** | |
| --- | --- | --- |
| **Year** | **ECT-course** | **M-ECT** |
| **2012*** | 9,9 | 7,9 |
| **2014** | 10,7 | 13,3 |
| **2015** | 10,9 | 12,8 |
| **2016** | 11,7 | 13,4 |
| **2017** | 11,4 | 14,4 |
| **2018** | 12,3 | 14,3 |
| **2019** | 12,4 | 15,3 |
| **2020** | 12,1 | 13,7 |
| **2021** | 12,2 | 15,4 |
| **2022** | 12,4 | 14,3 |

*Since the services of neuromodulation unit started in April 2012, the numbers cover a 9-month period from April to December 2012.
