# Supplementary material for: Change in the Use of Electroconvulsive Therapy Between 2012 and 2022 in Northern Finland
Source: J ECT. 2026 Jun 3;42(3):207–15. doi: 10.1097/YCT.0000000000001275 (PMC13561100; doi:10.1097/YCT.0000000000001275)
Supplement: Supplementary file 2 [file yct-42-207-s002.docx]

**Supplemental digital file Table 3.**

Main diagnostic categories of patients at the last ECT treatment episode during the whole study period from 2012-2022.

|  | **Unipolar depression (n=623)** | | **Bipolar disorder (n=86)** | | | **Psychotic disorders (n=221)** | | | **Other diagnoses** (n=57)** |
| --- | --- | --- | --- | --- | --- | --- | --- | --- | --- |
| **Year of last treatment episode** | **Non-psychotic** | **Psychotic** | **Non—psychotic**  **depression** | **Psychotic depression** | **Mania, hypomania** | **Schizophrenia** | **Schizo-affective** | **Other** |  |
|  | **F32.0–2, F33.0–2** | **F32.3, F33.3** | **F31.3–4** | **F31.5** | **F31.0–2** | **F20.0–9** | **F25.0–9** | **F22.0,**  **F23.9,**  **F29** |  |
| **2012** | 27 (50.0 %) | 8 (14.8 %) | 7 (13.0 %) | 2 (3.7 %) | 0 | 4 (7.4 %) | 2 (3.7 %) | 1 (1.9 %) | 3 (5.6 %) |
| **2013** | 36 (61.0 %) | 9 (15.3 %) | 2 (3.4 %) | 0 | 2 (3.4 %) | 4 (6.8 %) | 2 (3.4 %) | 0 | 4 (6.8 %) |
| **2014** | 28 (41.8 %) | 19 (28.4 %) | 7 (10.4 %) | 1 (1.5 %) | 0 | 6 (9 %) | 2 (3 %) | 2 (3.0 %) | 2 (3.0 %) |
| **2015** | 52 (55.9 %) | 14 (15.1 %) | 3 (3.2 %) | 0 | 3 (3.2 %) | 6 (6.5 %) | 5 (5.4 %) | 6 (6.5 %) | 4 (4.3 %) |
| **2016** | 52 (57.1 %) | 14 (15.4 %) | 6 (6.6 %) | 2 (2.2 %) | 1 (1.1 %) | 8 (8.8 %) | 2 (2.2 %) | 2 (2.2 %) | 4 (4.4 %) |
| **2017** | 38 (46.9 %) | 13 (16.0 5) | 5 (6.2 %) | 0 | 1 (1.2 %) | 12 (14.8 %) | 3 (3.7 %) | 3 (3.7 %) | 6 (7.4 %) |
| **2018** | 33 (41.8 %) | 12 (15.2 %) | 5 (6.3 %) | 0 | 0 | 9 (11.4 %) | 7 (8.9 %) | 9 (11.4%) | 4 (5.1 %) |
| **2019** | 30 (38.5 %) | 15 (19.2 %) | 9 (11.5 %) | 1 (1.3 %) | 1 (1.3 %) | 10 (12.8 %) | 7 (9.0 %) | 2 (2.6 %) | 3 (3.9 %) |
| **2020** | 44 (47.3 %) | 16 (17.2 %) | 2 (2.2 %) | 1 (1.1 %) | 1 (1.1 %) | 13 (14.0 %) | 6 (6.5 %) | 3 (3.2 %) | 7 (7.5 %) |
| **2021** | 43 (41.7 %) | 15 (14.6 %) | 6 (5.8 %) | 0 | 0 | 17 (16.5 %) | 10 (9.7 %) | 4 (3.9 %) | 8 (7.7 %) |
| **2022** | 70 (37.0 %) | 35 (18.5 %) | 13 (6.9 %) | 5 (2.6 %) | 0 | 31 (16.4 %) | 18 (9.5 %) | 5 (2.6 %) | 12 (6.3 %) |
| **Total*** | **453 (45.9 %)** | **170 (17.2 %)** | **65 (6.6 %)** | **12 (1.2 %)** | **9 (0.9 %)** | **120 (12.2 %)** | **64 (6.5 %)** | **37 (3.7 %)** | **57 (5.7 %)** |

*Diagnostic categories are defined based on the main diagnosis of a patient assessed at the last ECT visits during the whole study period between 2012-2022.

** includes diagnoses for OCD (ICD-10: F42.0-9), anxiety disorders (ICD-10: F40.0-9, F41.1-9), PTSD (ICD-10: F43.1) and somatoform disorders (ICD-10: F45.9) (n=24) and rest of diagnosis (n=33)
